# Editorial: Novel strategies for persistent atrial fibrillation ablation and AF driver mapping

**DOI:** 10.3389/fcvm.2023.1144723

**Published:** 2023-05-17

**Authors:** Mu Qin, Tao Liu, Shao-bo Shi, Shuang-lun Xie, Xiao-yan Qi

**Affiliations:** ^1^ Department of Cardiology, Shanghai Chest Hospital, Shanghai Jiaotong University, Shanghai, China; ^2^ Department of Cardiology, Renmin Hospital of Wuhan University, Wuhan, China; ^3^ Department of Cardiology, Sun Yat-sen Memorial Hospital, Sun Yat-sen University, Guangzhou, China; ^4^ Research Center, Montreal Heart Institute and Université de Montréal, Montreal, QC, Canada

**Keywords:** persistent atrial fibrillation, ablation strategy, driver, mapping, substrate

**Editorial on the Research Topic** Novel strategies for persistent atrial fibrillation ablation and AF driver mapping

Although pulmonary vein isolation (PVI) is an established and recommended catheter ablation approach for atrial fibrillation (AF), it yields suboptimal clinical outcomes in the setting of persistent AF due to the non-persistence of transmural lesions and unclear mechanisms of extra-pulmonary vein maintenance (1–3). Therefore, it is necessary to elucidate the underlying mechanisms and improve the success rate. The present research topic aims to explore new ablation strategies and mechanisms to increase the success rate of persistent atrial fibrillation, and includes a total of seven clinical studies.

Two studies in this topic focus on the transmural lesions of ablation. Cui et al. compared high-power (HP) vs. conventional-power (CP) radiofrequency ablation for AF and showed that HP ablation improved operation time and efficiency without increasing complications. HP ablation has gradually replaced CP ablation in various electrophysiological centers. Previous studies have shown that HP ablation is not inferior to CP ablation in terms of immediate effect and long-term success rate (4). The failure to achieve efficacy is mainly due to HP ablation shortening the ablation time, resulting in a decrease in the depth of damage (5). Therefore, on the other hand, HP ablation does not increase the risk of complications. However, the current research results indicate that the biggest advantage of HP ablation is to greatly improve ablation efficiency, and Cui et al.'s research has also reached a consistent conclusion. Additionally, a cardiac surgery study included in this topic used catheter mapping to test the transmural damage of MAZE IV surgery. This study found that the rate of incomplete bidirectional electrical isolation of the mitral isthmus ablation line or tricuspid annulus ablation line was 23.8%. Successful complementary ablation can improve the success rate of AF at 6 months. These findings suggest that even surgical ablation cannot ensure 100% linear blocking, and achieving a higher rate of transmural lesions can help maintain the sinus rate.

The cause of recurrent persistent AF (PersAF) has always been the focus and difficulty of clinical electrophysiological research. Vijayakumar et al. found that more than half of pre-ablation sources were repeated during post-ablation recurrence by the ECGI mapping system. 59% of the post-ablation AF drivers were mapped in the left atrium (LA), with 50% located around the pulmonary veins and up to 39% of sources mapped in the right atrium (RA). This study provides direct evidence for drivers that persist days and months after the ablation procedure, and bi-atrial ablation is necessary to improve the success rate of persistent AF.

Bi-atrial ablation has been shown to be effective in treating PersAF. In a study by Baptiste et al., it was found that the right atrium (RA) is involved in some of the maintenance mechanisms of PersAF. By conducting bi-atrial high-density mapping, the study found that 7% (22/317) of patients’ AF was driven by the right atrial appendage (RAA), and ablation allowed AF to reach intraoperative termination. RA has special arrhythmogenic structures such as RAA, superior vena cava, and terminal crest, which are important anatomical basis for the formation of functional reentry (6, 7). Therefore, when there is a short cycle-length potential in RAA (RAA firing) (8), it suggests that RA is involved in the maintenance of PersAF.

Substrate-based approaches have been the mainstream strategy for AF ablation, but whether substrate intervention is at sinus rate (SR) or in AF remains controversial. A study by Huang et al. found that the area of the low voltage region at SR is smaller than the Low voltage zone (LVZ) measured at AF. Prolonged fractionated potentials (PFP) at SR are also less than at AF. The SR-PFP distribution is consistent with LVZ, and there is a high degree of spatial agreement (80%) between PFP-AF and PFP-SR. Therefore, this study suggests that there is not much difference in the distribution area of LVZ between SR and AF, but substrate-based ablation at SR will reduce unnecessary myocardial damage.

Two research articles have studied the role and intervention effect of the atrial venous system in PersAF. Zhang et al. found that disconnection of the distal coronary sinus (CSD)-LA connection could reduce the inducible rate of acute AF and the recurrences of atrial arrhythmia during a 6-month follow-up. Kong et al. discussed the technical issues of Marshall ethanol infusion (EIVOM) and its long-term clinical outcomes for PersAF. In principle, both strategies can eliminate AF recurrence caused by CS-dependent micro-reentry AT. The VENUS study confirmed the effectiveness of EIVOM for PersAF (9), and it has been widely used. The main role of EIVOM is to reduce the recurrence of AF by increasing the mitral isthmus (MI) blocking rate and eliminating the AF trigger mechanism of VOM. However, the strategy still has technical issues, such as whether to perform ablation or EIVOM first, and the appropriate dosage for ethanol infusion. A previous study found that EIVOM first significantly reduced the number of radiofrequency applications needed to achieve MI block (10). Kong et al. found that the “EIVOM first” approach was associated with shorter procedural and MI ablation time than the “ablation first” approach. The ethanol dose >5.75 ml independently predicted a successful MI block. This study provides an important technical basis for the extensive development of EIVOM.

Overall, this research topic covers several studies on AF ablation strategies and techniques, offering insight into the challenging problem of treating PersAF. While there is still much work to be done in this field, these studies offer a glimmer of hope for improving clinical outcomes. It is our hope that this research topic will provide a valuable theoretical basis for future research on this important topic.
